# Target identification in *Fusobacterium nucleatum* by subtractive genomics approach and enrichment analysis of host-pathogen protein-protein interactions

**DOI:** 10.1186/s12866-016-0700-0

**Published:** 2016-05-12

**Authors:** Amit Kumar, Pragna Lakshmi Thotakura, Basant Kumar Tiwary, Ramadas Krishna

**Affiliations:** Centre for Bioinformatics, Pondicherry University, Puducherry, 605014 India; Centre Head, Centre for Bioinformatics, Pondicherry University, Puducherry, 605014 India

**Keywords:** *Fusobacterium nucleatum*, Colorectal cancer, Subtractive genomics approach, Therapeutic target proteins, Host-pathogen protein-protein interactions, Enrichment analysis, Functional annotation, DAVID, Gene ontology, Pathway and disease enrichment

## Abstract

**Background:**

*Fusobacterium nucleatum*, a well studied bacterium in periodontal diseases, appendicitis, gingivitis, osteomyelitis and pregnancy complications has recently gained attention due to its association with colorectal cancer (CRC) progression. Treatment with berberine was shown to reverse *F. nucleatum*-induced CRC progression in mice by balancing the growth of opportunistic pathogens in tumor microenvironment. Intestinal microbiota imbalance and the infections caused by *F. nucleatum* might be regulated by therapeutic intervention. Hence, we aimed to predict drug target proteins in *F. nucleatum*, through subtractive genomics approach and host-pathogen protein-protein interactions (HP-PPIs). We also carried out enrichment analysis of host interacting partners to hypothesize the possible mechanisms involved in CRC progression due to *F. nucleatum*.

**Results:**

In subtractive genomics approach, the essential, virulence and resistance related proteins were retrieved from RefSeq proteome of *F. nucleatum* by searching against Database of Essential Genes (DEG), Virulence Factor Database (VFDB) and Antibiotic Resistance Gene-ANNOTation (ARG-ANNOT) tool respectively. A subsequent hierarchical screening to identify non-human homologous, metabolic pathway-independent/pathway-specific and druggable proteins resulted in eight pathway-independent and 27 pathway-specific druggable targets. Co-aggregation of *F. nucleatum* with host induces proinflammatory gene expression thereby potentiates tumorigenesis. Hence, proteins from IBDsite, a database for inflammatory bowel disease (IBD) research and those involved in colorectal adenocarcinoma as interpreted from The Cancer Genome Atlas (TCGA) were retrieved to predict drug targets based on HP-PPIs with *F. nucleatum* proteome. Prediction of HP-PPIs exhibited 186 interactions contributed by 103 host and 76 bacterial proteins. Bacterial interacting partners were accounted as putative targets. And enrichment analysis of host interacting partners showed statistically enriched terms that were in positive correlation with CRC, atherosclerosis, cardiovascular, osteoporosis, Alzheimer’s and other diseases.

**Conclusion:**

Subtractive genomics analysis provided a set of target proteins suggested to be indispensable for survival and pathogenicity of *F. nucleatum*. These target proteins might be considered for designing potent inhibitors to abrogate *F. nucleatum* infections. From enrichment analysis, it was hypothesized that *F. nucleatum* infection might enhance CRC progression by simultaneously regulating multiple signaling cascades which could lead to up-regulation of proinflammatory responses, oncogenes, modulation of host immune defense mechanism and suppression of DNA repair system.

**Electronic supplementary material:**

The online version of this article (doi:10.1186/s12866-016-0700-0) contains supplementary material, which is available to authorized users.

## Background

*Fusobacterium nucleatum* is a gram-negative, anaerobic opportunistic pathogen from bacteroidaceae family that causes infection by adhering and invading into epithelial cells of mouth and gut, co-aggregating with other pathogenic bacteria and eukaryotic cells followed by modulating host immune responses [1]. *F. nucleatum* is known for its role in periodontitis, appendicitis, gingivitis and invasive infections of head, neck, lung, liver, heart and brain [2–6]. It is reported as an aetiological agent of osteomyelitis, particularly in head and neck region of the patients, who were already affected by chronic periodontitis or odontogenic abscess [7]. *F. nucleatum* can even pass through umbilical cord and causes pregnancy complications such as preterm birth, stillbirth and neonatal sepsis [4, 8]. Colonization by highly invasive *F. nucleatum* in intestine may be considered as useful biomarker for inflammatory bowel disease (IBD) diagnosis [2].

Whole genome and shotgun sequencing to characterize microbiota composition of colorectal cancer (CRC) tumor showed enriched DNA sequence of *F. nucleatum* ATCC 25586, suspecting its association with CRC [9, 10]. Subsequent studies on cell line and mouse tumor xenograft model showed that adhesion of *F. nucleatum* via FadA to E-cadherin on host cells increases CRC tumor growth by altering E-cadherin/β-catenin signaling and activating its downstream proinflammatory responses [11]. Accelerated colonic tumorigenesis due to *F. nucleatum* was also evidenced in adenomatous polyposis coli^+/−^ (APC^+/−^) mice by the generation of NF-kB-driven proinflammatory gene signature shared by human CRC [12]. Studies by Tahara et al., suggested its pathogenic role since *Fusobacterium* enrichment is associated with specific molecular subsets of CRC such as microsatellite instability (MSI), TP53 mutation, positivity of CpG island methylator phenotype (CIMP), hMLH1 methylation, CHD7/8 mutation [13]. In mice, *F. nucleatum* colonization alters intestinal microbial community by reducing symbiotic flora and increasing opportunistic bacteria and it also induces tumor related host immune cytokines. Both, the intestinal microbial structure and host immune defence plays essential role in CRC progression [14–17]. Berberine treatment was shown to reverse *F. nucleatum* mediated imbalance in luminal microbiota and thereby CRC growth in vivo studies [18]. Although no direct evidences available to characterize *F. nucleatum* as causative agent of CRC, these findings suggest the contribution of *F. nucleatum* enrichment to aggressiveness of CRC and its successive selection for designing therapeutic strategies. Thus, we focused on an *in silico* strategy to select target proteins in *F. nucleatum* which might be suitable for attenuating *Fusobacterium* pathogenicity with potent inhibitors.

Computational approaches in particular comparative and subtractive genomics have been extensively used to identify novel drug targets in infectious pathogens. These approaches are powerful, speedy and cost-effective in drug discovery and development processes compared to conventional methods. Taking this as an advantage, we implemented subtractive genomics approach [19] to predict drug targets in *F. nucleatum* which has fetched 33 druggable proteins altogether. Patients with long term IBD have an increased risk of CRC development [20]. Given the association between *F. nucleatum* infections and enhanced multiple gene expression in IBD and CRC, host-pathogen protein-protein interactions (HP-PPIs) were predicted to suggest putative targets and to hypothesize the plausible mechanisms in CRC progression provoked by *F. nucleatum* infection.

## Results and discussion

### Subtractive genomics approach

**Fig. 1:**
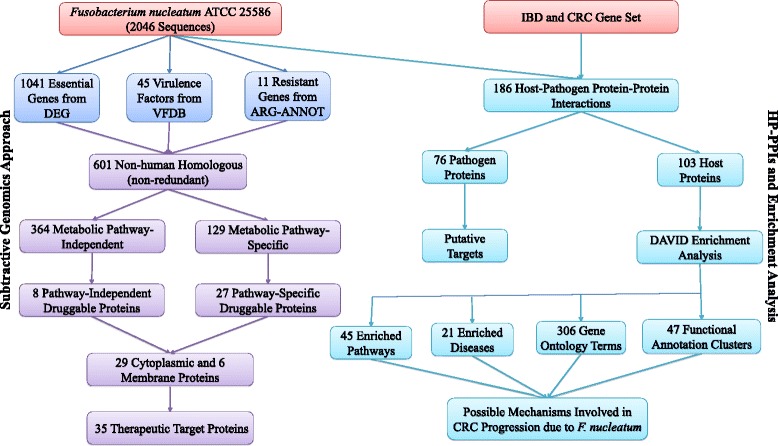
Schematic representation of workflow and the outcome of each step involved in computational subtractive genomics-based target identification in *F. nucleatum* and prediction of HP-PPIs between IBD, CRC dataset of host and *F. nucleatum*

The RefSeq proteome of *F. nucleatum* ATCC 25586 (NC_003454.faa) encompassing 2046 protein sequences was retrieved from NCBI bacterial genomes database. The schematic representation of workflow with number of permitted genes at each screening step is shown in Fig. 1.

### Essentiality analysis

Database of essential genes (DEG) consists of minimal gene set that is indispensable for cellular and organism viability [21]. A search for DEG homologous sequences in the proteome of *F. nucleatum* yielded 1041 hits (Additional file 1: Table S1) with cut-off e-value >1E-05 and bit score >100, representing their essentiality for bacterial survival. Manual cross-checking of each query protein’s biological function with its attained hit showed that they are similar in function. Obtained hits were found to be involved in structural organization, nutritional uptake, pathogenesis, antibiotic resistance and other essential processes for the survival of *F. nucleatum*.

### Analysis of Virulence Factors (VFs)

Genome sequence studies had reported putative VFs in *F. nucleatum* [22]. However, explored significance of this bacterium in various diseases necessitates the search for novel VFs. Thus, we attempted to predict VFs from whole proteome of *F. nucleatum* by searching against virulence factor database (VFDB) [23] as well as from literature reports of orthologous subspecies [24–27]. About 121 proteins of the whole proteome were found to have hits against VFDB with bit score >100 of which, 13 proteins alone were recognized as non-redundant to the essential proteins list obtained from DEG (Additional file 1: Table S2). Among them, the importance of few VFs in bacterial infection is discussed here (i) Hemolysin activation protein – lyses erythrocytes and creates anaerobic environment at the site of infection, attacks immune system of the host [28] (ii) Fibronectin-binding protein-like protein A – mediate adhesion of pathogen to host cells especially to fibronectin and elastin and helps in invasion [29] (iii) Choline kinase and Choline transport protein – catalyze phosphorylcholine attachment to carbohydrate, releasing CMP in lipopolysaccharide metabolism [22] (iv) Polysaccharide biosynthesis protein – could protect pathogen by producing resistance to complement-mediated killing by the host [30] (v) γ-polyglutamic acid synthetase – anchors to bacterial surface and helps in its survival in adverse conditions [31].

In addition, 114 VFs in *F. nucleatum* were identified from their homology to the respective proteins in orthologous subspecies such as *polymorphum* and *vincentii* [24, 32]. Among them, only 32 VFs were found to be non-redundant to the essential proteins list (Additional file 1: Table S2). These VFs include outer membrane proteins (OMPs), TraT complement resistance protein, transporter, secretion pathway proteins, serine proteases, periplasmic component of efflux system and other. OMPs of *F. nucleatum* not only mediate adherence with other pathogenic bacteria and host cells, but also suppress host immune system and induce cell death in lymphocytes thus, protecting tumor cells from immune cell attack [33]. TraT complement resistance protein and serine proteases also fight against host immune system and thus, enhances resistance of bacterium [34, 35].

### Resistance protein analysis

Resistance causing and drug efflux proteins could act as potential therapeutic targets [36]. Here, we obtained 118 antibiotic resistant genes in *F. nucleatum* with cut-off e-value of 1E-05 by employing Antibiotic Resistance Gene-ANNOTation (ARG-ANNOT) tool and from literature search of ortholog subsp. of *F. nucleatum.* The number of proteins was further narrowed down to 11, as 107 were observed either as essential or virulent (Additional file 1: Table S3). Some of the noteworthy proteins include acriflavin resistance proteins B, D, E, β-lactamase, multi-drug resistant protein, metal dependent hydrolase, macrolide-efflux protein and zinc metallohydrolase. Acriflavin resistance proteins B and E efflux multiple drugs directly to external surface and make the bacteria resistant. However, these proteins cannot efflux basic, hydrophilic aminoglycoside due to their hydrophobic cavity which is accomplished by homolog protein, AcrD. *F. nucleatum* also acquired resistance to β-lactam group of antibiotics like penicillin-G due to its ability to produce β-lactamases [37]. Genome annotation of five different strains of *Fusobacterium* also revealed the presence of multidrug resistance proteins and β-lactamases, which was consistent with our results obtained from ARG-ANNOT.

### Non-human homologous proteins

The search in *F. nucleatum* proteome mined 1097 non-redundant essential, virulence and resistance datasets. However, it is necessary to evaluate homology of these proteins in order to find out non-human homologous proteins and to design pathogen-specific inhibitors [38]. BLASTp search against proteome of *Homo sapiens* retrieved 601 non-homolog proteins out of which, only 237 were observed to be involved in 70 metabolic pathways of *F. nucleatum* according to Kyoto Encyclopedia of Genes and Genomes (KEGG) mapper (Additional file 2: Table S4 and S5). The remaining 364 pathway-independent proteins were directly considered for druggability analysis to understand their therapeutic potential.

### Differential pathway analysis

Manual comparison between the enlisted 70 metabolic pathways of *F. nucleatum* and all 278 metabolic pathways of human (present in KEGG) resulted in only 18 pathways that are specific to *F. nucleatum*. These pathways were found to possess only 96 non-redundant proteins (Additional file 2: Table S6).

### Druggability analysis

A protein is considered as ‘druggable target’ only when binding of small molecule either drug/vaccine modulates its function with beneficiary effects to the host [39].

### Druggability of pathway-independent proteins

The 364 proteins that do not belong to any metabolic pathway of *F. nucleatum* were found to play a key role in pathway-independent processes such as prokaryotic cytokinesis, drug efflux, DNA replication, repair, transcription, translation and colonization in host. Hence, we considered them for druggability screening which resulted in only eight proteins with therapeutic characteristics. They are acetoacetate metabolism regulatory protein atoC, acriflavin resistance protein B, cell division protein FtsZ, flavodoxin, pseudouridine synthase, putative ribosome biogenesis GTPase RsgA, putative tRNA (cytidine(34)-2’-O)-methyltransferase and RNA polymerase sigma factor SigA. Functional significance of each of these proteins is mentioned in Table 1.

**Table 1:** Metabolic pathway-independent druggable proteins

S. No.	Gene	Protein name	Uniprot ID	Function
1	FN1275	Acriflavin resistance protein B	Q8RE51	Resistant to aminoglycosides such as amikacin, gentamycin, kanamycin, neomycin and tobromycin as well as to amphiphilic compounds. Thus, inhibiting AcrB function may lead to pathogen’s susceptibility to these antibiotics.
2	FN1321	Acetoacetate metabolism regulatory protein atoC	Q8RE11	Required for catabolism of short-chain fatty acids. Plays role in flagella synthesis, sodium (but not potassium) sensitivity and chemotaxis.
3	ftsZ	Cell division protein FtsZ	Q8RDQ7	Required for proper localization of division plane during bacterial cytokinesis. Inhibition of its GTP-dependent polymerization activity prevents cells to divide
4	FN0724	Flavodoxin	Q8RFH4	Acts as an electron acceptor of pyruvate-oxidoreductase complex. Flavodoxin inhibitors were reported to act against *Helicobacter pylori*’s infection that causes gastritis or chronic peptic ulcers
5	FN0756	Pseudouridine synthase	Q8RFE9	Catalyze post-transcriptional modifications of cellular RNAs i.e., site-specific isomerisation of uridine.
6	rsgA	Putative ribosome biogenesis GTPase RsgA	Q8R685	Involved in 30S ribosome subunit biogenesis.
7	FN0809	Putative tRNA (cytidine(34)-2’-O)-methyltransferase	Q8R673	Methylates 2’-O-ribose group of cytidine present at 34^th^position of tRNA anticodon loop.
8	sigA	RNA polymerase sigma factor SigA	Q8RE13	Essential for survival of bacteria as it regulates transcription of housekeeping promoters.

### Druggability of pathway dependent proteins

From 96 pathogen-specific metabolic pathway proteins, only 27 proteins involved in 15 pathways were observed to be druggable in nature. Segregation of these 27 proteins into their metabolic pathways revealed that 12 of them belong to peptidoglycan biosynthesis pathway among which, six were also shared by vancomycin resistance and beta-lactam resistance pathways (3 in each). Further, lipopolysaccharide biosynthesis pathway and microbial metabolism in diverse environments had five druggable proteins in each (shared by CAMP resistance pathway, propionate metabolism, D-alanine metabolism). Rest of the pathways possessed only one druggable target protein in each (Table 2). Thus, this study suggested the crucial pathways for survival of *F. nucleatum*, which can be targeted for drug discovery in pathway centric approach.

**Table 2:** Druggable proteins and their metabolic pathways along with accession number

S. No.	Uniprot ID	No. of Proteins	KEGG pathway	KEGG ID
1	Q8RIQ1, Q8RDQ3, Q8RDQ2, Q8RDQ1, Q8R635, Q8R5N5, Q8RDP8, Q8RDQ4, Q8RG00, Q8RFV2, Q8REF2, Q8REA2	12	Peptidoglycan biosynthesis	fnu00550
2	Q8RFU2, Q8R691, Q8R6A2, Q8RE91, Q8RFA8	5	Lipopolysaccharide biosynthesis	fnu00540
3	Q8RFB7, Q8RED6, Q8R612, Q8R609, Q8RHW6	5	Microbial metabolism in diverse environments	fnu01120
4	Q8RGA2, Q8RDQ4, Q8R5N5, Q8RDP8	4	Vancomycin resistance	fnu01502
5	Q8RG00, Q8REF2, Q8REA2	3	beta-Lactam resistance	fnu01501
6	Q8R612, Q8RHW6	2	D-Alanine metabolism	fnu00473
7	Q8R612	1	Chloroalkane and chloroalkene degradation	fnu00625
8	Q8RED6	1	Propanoate metabolism	fnu00640
9	Q8R612	1	Butanoate metabolism	fnu00650
10	Q8RHW6	1	Biosynthesis of secondary metabolites	fnu01110
11	Q8RFU2	1	Cationic antimicrobial peptide (CAMP) resistance	fnu01503
12	Q8RGT8	1	Two-component system	fnu02020
13	Q8REE2	1	Bacterial chemotaxis	fnu02030
14	Q8RI43	1	Phosphotransferase system (PTS)	fnu02060
15	Q8RHE2	1	Bacterial secretion system	fnu03070

### Peptidoglycan biosynthesis pathway

From the current study, MurA to MurG proteins, D-alanine--D-alanine ligase (Ddl), penicillin-binding protein (PBP), cell division protein ftsI of peptidoglycan biosynthesis pathway were predicted as target proteins in *F. nucleatum*. MurA catalyzes the first committed step of this pathway where it converts UDP-N-acetylglucosamine (aminosugar) to UDP-N-acetylglucosamine enolpyruvate. Targeting this protein could be lethal to bacterium as *F. nucleatum* contains only one copy of MurA [40]. MurB is also an attractive target as it catalyzes the reduction of UNAG-enolpyruvate [41]. MurC, D, E and F ligases sequentially attach L-alanine, D-glutamate, 2, 6-diaminopimelate and D-alanyl-D-alanine to form UDP-MurNAc-peptide [42]. Bacteria acquire vancomycin resistance if MurF joins D-Ala-D-Lac or D-Ala-D-Ser to UDP-MurNAc-tripeptide instead of D-Ala-D-Ala [43]. MurG catalyzes glycosyl transfer from UDP-GlcNAc to MurNAc present in lipid I to produce lipid II [44]. Penicillin binding proteins (PBPs) translocate this lipid II from cytoplasm to exterior surface of the cell and incorporate into peptidoglycan layer [45]. Thus, PBP is also an important target protein for inhibitor development [46]. These proteins are not only essential for bacterial survival, but also involve in the pathogenesis by interacting with host cytoskeleton [47]. Hence, targeting this pathway could lead to bacterial lysis by weakening the rigidity and strength of cell wall. Moreover, lack of homologous counterpart proteins in human make them an excellent drug targets.

### Lipopolysaccharide (LPS) biosynthesis pathway

In LPS biosynthesis pathway, lpxA, lpxC, KdsA, KdsB and gmhA were considered as valid targets for drug development. Enzymes lpxA and lpxC (catalyzes committed step) contribute to the formation of lipid A which is a conserved core oligosaccharide region of LPS molecules. Lipid A is responsible for toxic effects of gram-negative bacterial infection. The target protein KdsB (CMP-Kdo synthetase) is unique in its direct coupling of KDO (sugar) to cytosine monophosphate (CMP) in KDO biosynthesis which is a part of LPS inner core. Another druggable protein obtained in this pathway is gmhA (lpcA) that catalyzes the first step in biosynthesis of ADP-L-glycero-D-manno heptose [48]. Inhibiting gmhA would create heptose less core LPS leading to enhanced sensitivity of *F. nucleatum* to even low concentration of novobiocin, detergents and bile salts [49].

### Subcellular localization

Prediction of subcellular localization of druggable targets using various online servers delineated that 29 out of 35 target proteins were located in cytoplasm while the rest were in membrane (Additional file 2: Table S7). Cytoplasmic proteins can be considered for small molecule drug development while membrane or secreted proteins for vaccine development.

### Host-pathogen protein-protein interactions

**Fig. 2:**
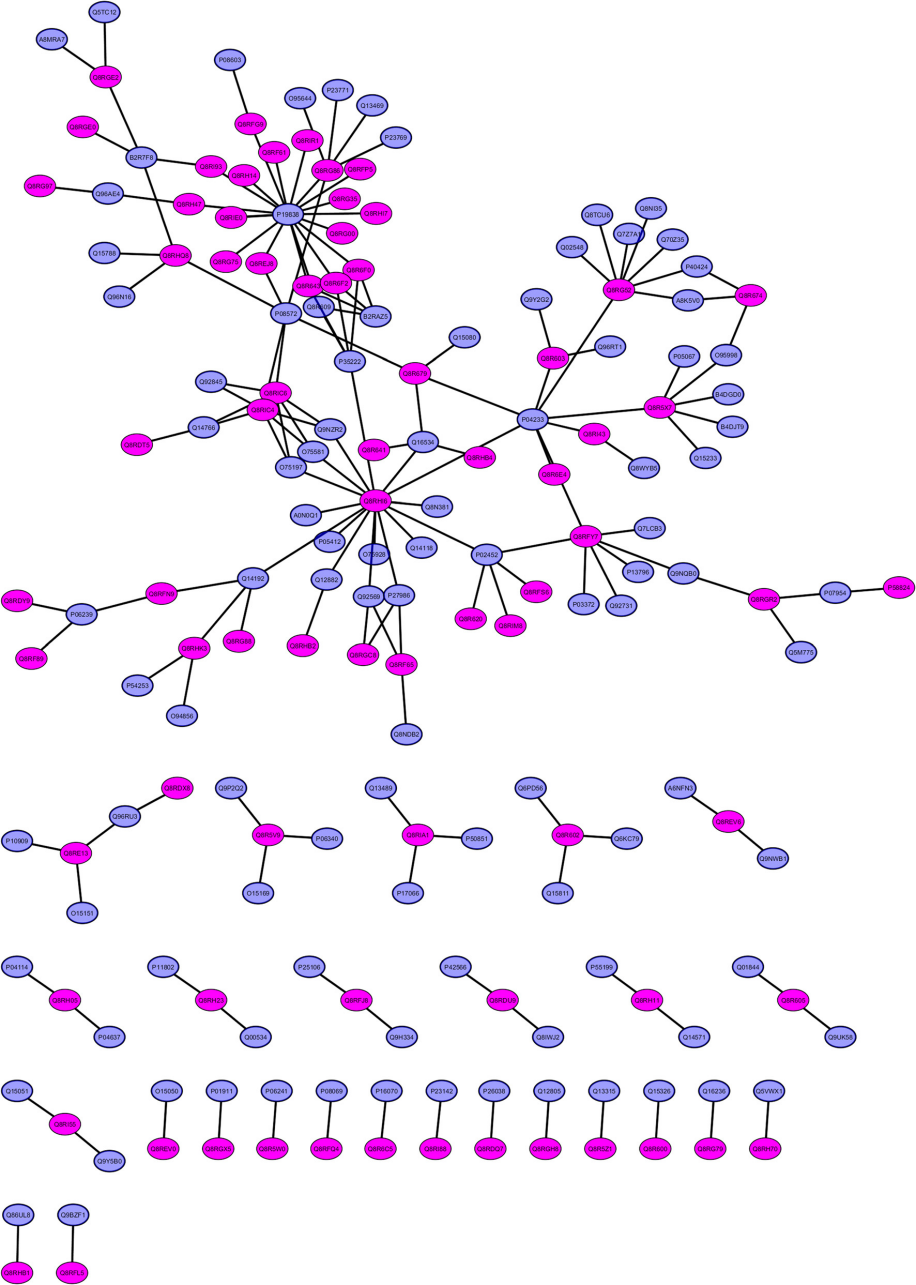
Biological network of host-pathogen protein-protein interactions are shown using Cytoscape. *F. nucleatum* and host interacting proteins are represented in pink and blue color respectively

As *F. nucleatum* infects the host through its adherence and invasiveness, insights into HP-PPIs could help us to understand the mechanism of infection, disease establishment and to identify putative drug targets. The experimentally verified HP-PPIs information is currently limited for *F. nucleatum*. In our attempt to predict the homology based HP-PPIs between *F. nucleatum* proteome and IBD, CRC related protein dataset of human using HPIDB yielded 186 interactions, participated by 103 host and 76 pathogenic proteins (Additional file 3: Table S8). Predicted interactions were based on homologous HPIs detected by experimental methods such as two-hybrid pooling approach, surface plasmon resonance, affinity chromatography, far Western blotting, anti-tag co-immunoprecipitation and nuclear magnetic resonance in *Bacillus anthracis*, *Yersinia pestis*, *Francisella tularensis* subsp. *tularensis* SCHU s4, *Saccharomyces cerevisiae* S288c, *Escherichia coli* K-12 and *E. coli* O157:H7 organisms collected from IntAct, MINT, BIOGRID, DIP databases. The biological network of predicted HP-PPIs is presented in Fig. 2. From these HP-PPIs, the *F. nucleatum* interacting partners were reported as putative targets while host interacting partners were validated by enrichment analysis to predict their biological processes.

### Enrichment analysis of host proteins

If a set of proteins are significantly enriched in certain biological processes or pathways, they are likely to play similar role in vivo [50]. Hence, pathway, functional, disease enrichment analysis and functional annotation clustering were carried out to assess predicted HP-PPIs using Database for Annotation, Visualization and Integrated Discovery (DAVID) [51]. This analysis resulted in significantly enriched 45 pathways, 306 gene ontology (GO) terms and 21 diseases with minimum count of three gene set and p-value lower than 0.05 in addition to 47 annotation clusters with cut-off enrichment score of > 1 and minimum of three terms.

### Pathway enrichment analysis

This analysis was performed based on the assessment that host proteins involved in HPIs possess functional correlation to the pathways that are relevant to pathogen’s infection. Among the obtained pathways from DAVID Functional Annotation Chart analysis, Wnt signaling pathway, ECM-receptor interactions, focal adhesion, toll-like receptor (TLR) signaling and CRC probably suggest the role of *F. nucleatum* in adhesion, invasion into epithelial cells and triggering of signal transduction pathways that promote CRC [1, 11, 26, 52]. Other attained pathways such as T-cell, B-cell receptor signaling, T-cell, B-cell activation, antigen processing and presentation, Lck and Fyn tyrosine kinases in initiation of TCR activation, viral myocarditis indicate the relevance of *F. nucleatum* infection to host immune responses [11, 53–55]. *F. nucleatum* infection inhibits T-cell activation by preventing cells to exit G0/G1 phase of cell cycle which may result in local and/or systemic immunosuppression [53]. Chronic inflammation in periodontal diseases due to *F. nucleatum* has been reported as potential risk factor for Alzheimer’s disease [56]. Most of the obtained pathways were in accordance to literature reports while the rest of them could be either false positive or may have an indirect effect which seeks further experimental support. This include renal cell, basal cell, small cell lung, non-small cell lung, endometrial, thyroid, pancreatic, acute myeloid leukemia, glioma, leukocyte transendothelial migration and melanoma.

### Functional and disease enrichment

Similar to pathway enrichment analysis, GO term analysis of host interacting partners also showed the involvement of *F. nucleatum* in Wnt signaling pathway through β-catenin, T & B cell activation, immune system development, cell differentiation, cytoskeleton and apoptosis. GO term analysis also added other processes including cell proliferation, cell motion, membrane invagination, cell cycle control, organ development, tissue cell and organ morphogenesis, receptor & lipid binding, blood vessel development, homeostasis process, embryogenesis, neurogenesis and others which were also supported by literature (Additional file 3: Table S10) [26, 27, 53, 55, 57–60].

Disease enrichment analysis of host interacting partners showed the association of *F. nucleatum* with CRC, atherosclerosis, cardiovascular diseases, blood pressure, arterial hypertension, Alzheimer’s and osteoporosis (Additional file 3: Table S11) [56, 61–63]. Systemic inflammation and immune cross-reactivity due to *F. nucleatum* infection play a major role in atherogenesis [62] and may increase the risk to develop atherosclerotic cardiovascular diseases, blood pressure [64]. *F. nucleatum* may play a role in osteoporosis as it is a strong inducer of osteoclast activity and stimulates bone resorption [65]. Other diseases such as endometrial, ovarian cancer, melanoma could be either false positive or may share common signaling pathways with other diseases of *F. nucleatum*.

In order to further validate the results, we performed Functional Annotation Clustering analysis which yielded 47 functional clusters (Additional file 3: Table S12). Most over-represented terms in this functional cluster analysis were related to focal adhesion, immune responses, cell cycle, cell differentiation & death, wnt, p53 pathways and positive regulation of transcription.

**Fig. 3:**
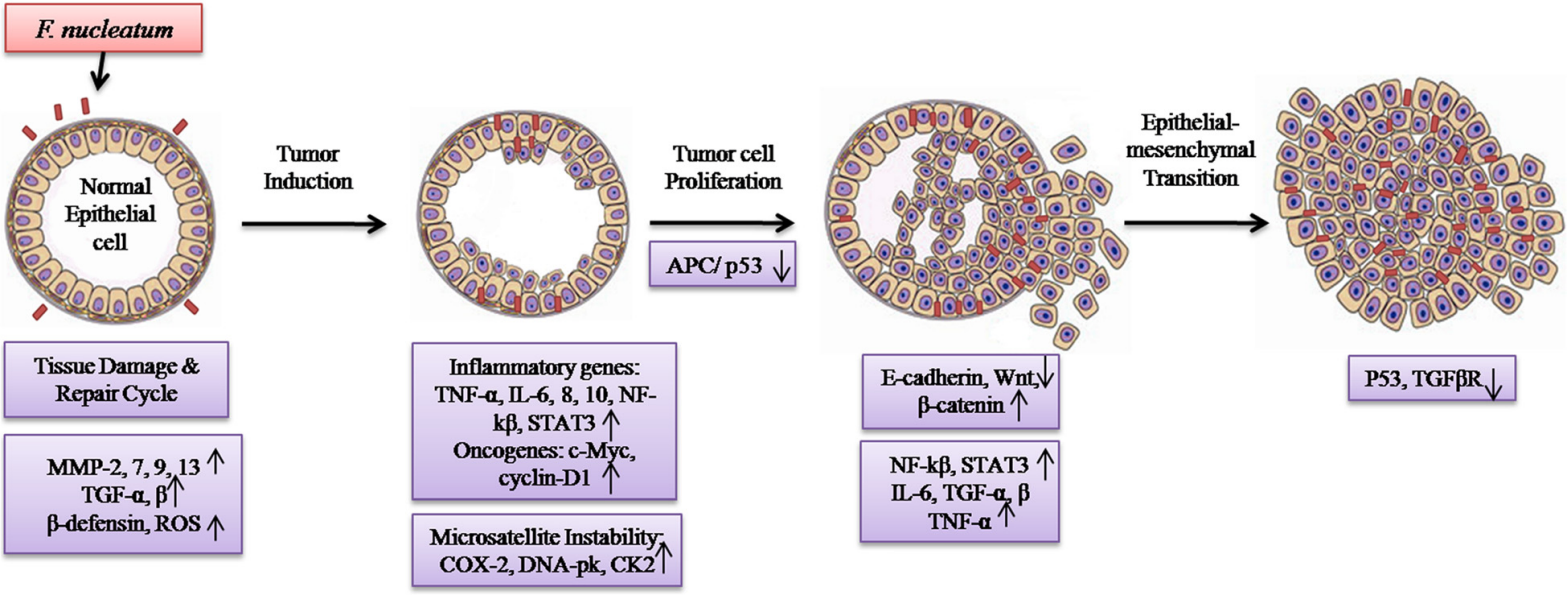
The plausible mechanisms in CRC progression due to *F. nucleatum* infection may involve increased expression of proinflammatory genes, ROS, oncogenes, DNA damaging genes and β-catenin signaling which may contribute to accumulation of mutations that promote tumor induction, proliferation and epithelial-mesenchymal transition

Based on this enrichment analysis, we hypothesized the possible mechanisms involved in CRC progression due to *F. nucleatum* infection (Fig. 3). Host epithelial cells detect *F. nucleatum* infection via TLRs [52]. Infection by this bacterium damages the gut tissue by inducing NF-kB driven overexpression of inflammatory genes such as collagenase-3, IL-6, 8, 10, TGF-α, TGF-β (through p38 MAPK activation) and TNF-α [26]. Responding to this damage, epithelial cells stimulate repair and defensive (wound healing) mechanism by producing antimicrobial peptide, β-defensins [55]. However, the bacterium escapes from antimicrobial effects of host and persists inside epithelial cells possibly by interfering with phagosome-endosome system [26]. Thus, the cycle of tissue damage and repair mechanism continues which might promote tumor induction. Besides, overexpression of proinflammatory genes could also facilitate tumor invasion and extravasation by regulating the production and activity of extra cellular matrix degrading proteases. Proinflammatory genes may enhance the expression of anti-apoptotic (Bcl2, Bcl-xL), proliferative genes (c-Myc, cyclin D1) and pro-angiogenesis factor (VEGF) via STAT3 up-regulation. Localized inflammatory microenvironment created by up-regulated inflammatory genes and oxidative damage by ROS possibly induces DNA mutations and epigenetic changes in host cells. However, host DNA repair mechanism unable to restore this damage as *F. nucleatum* induces the production of kinases involved in DNA damage (DNA-PK), death-associated protein kinase 1, protein kinase CK-2 and COX-2. This results in accumulation of oncogenic or inactivating mutations in tumor suppressor genes such as p53 and APC. During *F. nucleatum* adhesion, downregulated expression of E-cadherin by FadA binding could lead to epithelial-mesenchymal transition (EMT) due to enhanced expression of β-catenin-regulated transcription factors such as lymphoid enhancer factor (LEF)/T-cell factor (TCF), NF-kB, Myc and Cyclin D. Thus, chronic infection of *F. nucleatum* may alter local cytokine profile, DNA repair system, apoptotic, proliferative and immune responses in infected cells and modulates the microenvironment of gut towards CRC.

## Conclusion

A set of proteins that were proposed through this computational approach might act as suitable targets for therapeutic intervention of *F. nucleatum*. In future, potent inhibitors can be designed to these predicted targets followed by an experimental verification. This might help in treatment of periodontal or other diseases caused by *F. nucleatum* and to reverse *F. nucleatum*-induced intestinal microbial imbalance in CRC progression. HP-PPIs predicted from this study might help in further mechanistic and functional exploration of *F. nucleatum* infection. Pathway, functional and disease enrichment analysis corroborated that predicted HP-PPIs were closely correspond to *F. nucleatum* infection. And the enrichment analysis of host interacting partners suggested that this bacterium might enhance CRC progression by affecting multiple signal transduction pathways, immune responses and tumor suppressive genes. Thus this study revealed the possible target proteins in *F. nucleatum*, predicted host-pathogen interacting partners and hypothesized the mechanisms involved in CRC progression due to infection of *F. nucleatum* which altogether may be applied for preventive or complementary therapy against *F. nucleatum*.

## Methods

The RefSeq proteome of *F. nucleatum* was downloaded from NCBI ftp for subtractive genomics analysis and to predict HP-PPIs.

### Subtractive genomics approach

#### Essential proteins

DEG consists of experimentally identified 22,343 essential protein-coding genes, 646 non-coding RNAs, promoters, regulatory sequences, and replication origins from 31 prokaryotes and ten eukaryotes [21, 66]. The queried genes having homologous hit in DEG are likely to be essential. Thus, BLASTp search was performed for the proteome of *F. nucleatum* against DEG bacterial proteins with cut-off parameters of 1E-05 e-value and bit score of 100. Query proteins and their corresponding hits were then manually compared for similar biological function. Proteins that satisfy these criteria alone were considered essential for bacterial survival.

### Virulence factors

VFs are inherent properties of pathogen which help the bacteria to adhere, colonize, invade, conquer host defence mechanism and cause disease. Understanding potential VFs from *F. nucleatum* and inhibiting them would make the pathogen non-virulent. VFDB, a comprehensive database that consists of four categories of VFs namely offensive, defensive, non-specific and virulence-associated regulated genes from 25 pathogenic bacteria [23]. The proteome of *F. nucleatum* was subjected to BLASTp search against database of protein sequences from VFDB core dataset (R1) with default parameters. The query sequences having hit with cut-off bit score >100 were only considered as VFs. Additionally, the literature reported VFs were collected from closely related subspecies of *F. nucleatum* including *polymorphum* and *vincentii.* Homology search of these proteins was performed to find exact matches in *F. nucleatum* subsp. *nucleatum* and thus constituted the list of VFs.

### Resistance proteins

Antibiotic therapy is generally used to treat *F. nucleatum* infections [67]. However, the bacterium has acquired resistance to several antibiotics by not only synthesizing β-lactamase but also by increased production of efflux proteins [68]. It has also showed resistance to human β-defensins (antimicrobial peptides) [25] and IgA due to which there is significant gain in its adherence to mucosal surfaces and colonization [69, 70]. Thus it is obligatory to study resistant proteins in *F. nucleatum* to prevent its infections*.* We implemented ARG-ANNOT tool [71] and literature search in ortholog subsps of *F. nucleatum* to identify existing and putative new antibiotic resistant genes in *F. nucleatum*. ARG-ANNOT contains 1689 antibiotic resistant amino acid sequences from various classes including aminoglycosides, beta-lactamases, fosfomycin, fluoroquinolones, glycopeptides, macrolide-lincosamidestreptogramin, phenicols, rifampicin, sulfonamides, tetracyclines and trimethoprim. A local BLAST program was run for the proteome of *F. nucleatum* in Bio-Edit software against antibiotic resistant sequences in ARG-ANNOT with cut-off e-value of 1E-05.

### Non-homologous proteins to host

BLASTp search of comprised list from the above three independent searches was performed against non-redundant protein sequence (nr) database of the host *Homo sapiens* (taxid: 9606), with an expected threshold value of 0.005. Proteins that did not show significant homology were only considered as non-homologous to human. These were sorted into their respective metabolic pathways to select pathogen specific pathways. Proteins that do not belong to any of metabolic pathways were directly considered for druggability screening.

### Pathogen specific pathways

KEGG pathway database [72] was used to find out the metabolic pathways of non-homologous proteins of *F. nucleatum*. Similarly, host metabolic pathways were also enlisted along with their accession number. The enlisted host and pathogen metabolic pathways were manually compared to identify the pathways that present only in the pathogen but not in the host *H. sapiens.*

### Druggable proteins

Druggability of screened proteins was investigated against all drug targets present in DrugBank database [73] and Therapeutic Targets Database (TTD) [74] respectively. Drugbank contains 4323 non-redundant protein sequences including drug target/enzyme/transporter/carrier linked to 6712 drug entries consisting of 1448 FDA-approved small molecule drugs, 131 FDA-approved biotech (protein/peptide) drugs, 85 nutraceuticals and 5080 experimental drugs. While TTD contains 2025 targets, including 364 successful, 286 clinical trial, 44 discontinued and 1331 research targets. The resultant hits from these two searches with bit score >100 and e-value <0.005 were considered as potential therapeutic candidates. Biological location of druggable proteins was classified as either cytoplasmic or membrane proteins based on the consensus location predicted using various online servers including PSORTb [75], Secretome [76], Cell-PLoc [77], CelloGram [78], PSLpred [79], and SOSUI [80].

### Host-pathogen protein-protein interactions

HP-PPIs are rich in information to understand molecular mechanism of pathogenicity. In the current work, we investigated HP-PPIs of *F. nucleatum* with respect to its infection in IBD and CRC. The RefSeq proteome of pathogen and host gene set was subjected to homology-based approach [81] implemented in Host-Pathogen Interaction DabaBase (HPIDB) which integrates experimental Protein-Protein Interactions (PPIs) from several public databases such as IntAct, GeneRif, Bind, Patric, VirHostNet, BioGRID, Reactome and MINT. HPIDB predicts HP-PPIs by serving homologous experimentally derived HP-PPIs as template [82]. The resultant HP-PPIs were scrutinized manually for similar biological function with their corresponding homologous and experimentally validated HP-PPI template given in the output.

### Pathway and functional enrichment analysis

The pathway, functional enrichment analysis and gene-annotation are promising approaches to identify the most significant and relevant biological processes of large gene list. Host genes that were predicted to interact with *F. nucleatum* were validated by Functional Annotation Clustering tool in DAVID v6.7 [50, 83]. DAVID provides statistically enriched pathways, diseases and GO terms for input genes using biological information present in pathway (BBID, BIOCARTA, KEGG, PANTHER and REACTOME), disease (OMIM and Genetic Association Disease) databases and Gene Ontology Consortium. The enrichment analytic algorithm used in this tool performs batch annotation and provides various annotation categories including GO terms, disease and pathway associations, protein-protein interactions and others. We have annotated an individual chart report of host gene list for GO terms, disease associations and bio-pathways to highlight statistically significant enriched terms with count threshold of 3 and EASE threshold of 0.1. Functional Annotation Clustering of host gene list with default databases was done to cluster a group of terms with similar biological function. It implies kappa statistics and fuzzy heuristics to cluster the data into an organized biological term modules and calculates the enrichment scores, *p*-value (Fisher’s Exact) with Benjamini correction. The terms with minimum gene set of 3 and cut-off p-value of 0.05 and clusters with enrichment score >1.0 were considered significant.

### Ethics approval and consent to participate

Not applicable.

### Availability of data and material

The data and materials supporting the conclusions of this article are included within the article and its additional files.

## Additional files

Additional file 1: Table S1. List of essential genes in *F. nucleatum* from DEG. **Table S2.** virulence factors list from VFDB search and literature. **Table S3.** List of resistance causing proteins from ARG-ANNOT tool. (XLSX 66 kb) Additional file 2: Table S4. Non-human homologous proteins present in *F. nucleatum*. **Table S5.** Metabolic pathways of non-human homologous proteins according to KEGG. **Table S6.** Pathogen-specific metabolic pathways of screened proteins in *F. nucleatum*. **Table S7.** Subcellular localization of druggable proteins. (XLSX 45 kb) Additional file 3: Table S8. Homology based HP-PPIs predicted from HPIDB. **Table S9.** Pathway enrichment analysis of host genes from DAVID functional annotation tool. **Table S10.** Gene ontology report of host genes. **Table S11.** Disease enrichment analysis of host genes participated in HP-PPIs. **Table S12.** Total functional annotation cluster analysis of host genes. (XLSX 99 kb)

## Abbreviations

APC: adenomatous polyposis coli
ARG-ANNOT: antibiotic resistance gene-ANNOTation
CRC: colorectal cancer
DAVID: the database for annotation, visualization and integrated discovery
DEG: database of essential genes
EMT: epithelial-mesenchymal transition
GO: gene ontology
HPIDB: host-pathogen interaction database
HP-PPIs: host-pathogen protein-protein interactions
IBD: inflammatory bowel disease
KEGG: kyoto encyclopedia of genes and genomes
LEF: lymphoid enhancer factor
LPS: lipopolysaccharide
OMPs: outer membrane proteins
RND: resistance-nodulation-cell division
TCGA: The Cancer Genome Atlas
TLRs: toll-like receptors
VFDB: virulence factors database
